# Supplementary material for: Comparison of Motor Scores between OFF and ON States in Tremor-Dominant Parkinson’s Disease after MRgFUS Treatment
Source: J Clin Med. 2022 Aug 2;11(15):4502. doi: 10.3390/jcm11154502 (PMC9369361; doi:10.3390/jcm11154502)
Supplement: Supplementary file 1 [file jcm-11-04502-s001.zip › jcm-1762182-supplementary.pdf]

Table S1. The medication of nine patients pre- and post-operation.

Patient Id	Pre-Operation	1 Month Post-Operation	3 Months Post-Operation	1 Year Post-Operation
429005	Madopar 125 mg qid Pramipexole dihydrochloride 0.125 mg bid	Madopar 125 mg tid	Madopar 125 mg tid	Madopar 125 mg tid
429003	Madopar 250 mg tid Pramipexole dihydrochloride 0.25 mg tid	Madopar 250 mg tid	Madopar 250 mg;125 mg;125 mg Pramipexole dihydrochloride 0.25 mg tid	Madopar 250 mg;187.5 mg;187.5 mg Pramipexole dihydrochloride 0.25 mg tid
429009	Madopar 62.5 mg tid Pramipexole dihydrochloride 0.5 mg tid	Madopar 125 mg tid Pramipexole dihydrochloride 0.25 mg tid	Madopar 125 mg tid Pramipexole dihydrochloride 0.25 mg tid	Madopar 187.5 mg;125 mg;125 mg Pramipexole dihydrochloride 0.25 mg tid
429006	Madopar 250 mg tid Selegiline 5 mg bid	Madopar 125 mg tid	Madopar 125 mg tid Pramipexole dihydrochloride 0.25 mg bid selegiline 5 mg qd	Madopar 125 mg tid Pramipexole dihydrochloride 0.5 mg bid
429012	Madopar 187.5 mg tid Pramipexole dihydrochloride 0.125 mg bid	Madopar 187.5 mg tid Pramipexole dihydrochloride 0.125 mg bid	Madopar 187.5 mg tid Pramipexole dihydrochloride 0.125 mg bid	Madopar 125 mg tid Selegiline 5 mg bid
429008	Madopar 187.5 mg tid	Madopar 187.5 mg tid	Madopar 187.5 mg tid	Madopar 187.5 mg tid
429010	Madopar 125 mg tid Trihexyphenidyl 2 mg tid	Madopar 125 mg tid Trihexyphenidyl 2 mg tid	Madopar 125 mg qid adamantanamine 100 mg bid	Madopar 250 mg; 125 mg;250 mg Trihexyphenidyl 2 mg tid Adamantanamine 100 mg bid
429013	Madopar 250 mg tid	Madopar 250 mg tid	Madopar 250 mg tid	Madopar 250 mg tid
429011	Madopar 250 mg tid	Madopar 250 mg tid	Madopar 250 mg tid	Madopar 250 mg tid
